# Co-occurrence and Mutual Exclusivity Analysis of DNA Methylation Reveals Distinct Subtypes in Multiple Cancers

**DOI:** 10.3389/fcell.2020.00020

**Published:** 2020-01-29

**Authors:** Wubin Ding, Guoshuang Feng, Yige Hu, Geng Chen, Tieliu Shi

**Affiliations:** ^1^Center for Bioinformatics and Computational Biology, Institute of Biomedical Sciences, School of Life Sciences, East China Normal University, Shanghai, China; ^2^Big Data and Engineering Research Center, Beijing Pediatric Research Institute, Beijing Children’s Hospital, Capital Medical University, National Center for Children’s Health, Beijing, China; ^3^Biological Targeting Diagnosis and Therapy Research Center, Guangxi Medical University, Nanning, China

**Keywords:** DNA methylation, co-methylation, mutual exclusivity, survival analysis, pan-cancer, cancer diagnosis

## Abstract

Co-occurrence and mutual exclusivity (COME) of DNA methylation refer to two or more genes that tend to be positively or negatively correlated in DNA methylation among different samples. Although COME of gene mutations in pan-cancer have been well explored, little is known about the COME of DNA methylation in pan-cancer. Here, we systematically explored the COME of DNA methylation profile in diverse human cancer. A total of 5,128,332 COME events were identified in 14 main cancers types in The Cancer Genome Atlas (TCGA). We also identified functional epigenetic modules of the zinc finger gene family in six cancer types by integrating the gene expression and DNA methylation data and the frequently occurred COME network. Interestingly, most of the genes in those functional epigenetic modules are epigenetically repressed. Strikingly, those frequently occurred COME events could be used to classify the patients into several subtypes with significant different clinical outcomes in six cancers as well as pan-cancer (*p*-value ≤ = 0.05). Moreover, we observed significant associations between different COME subtypes and clinical features (e.g., age, gender, histological type, neoplasm histologic grade, and pathologic stage) in distinct cancers. Taken together, we identified millions of COME events of DNA methylation in pan-cancer and detected functional epigenetic COME events that could separate tumor patients into different subtypes, which may benefit the diagnosis and prognosis of pan-cancer.

## Introduction

DNA methylation (DNAm) is a major epigenetic modification, which is considered as an approach for disease diagnosis. An increasing number of studies have indicated that aberrant DNAm plays an important role in diverse diseases, especially cancers (Delpu et al., 2013; Stefansson et al., 2015; Tahara and Arisawa, 2015). For example, the hypermethylation of CpG island in promoter region of tumor suppressor genes have been observed in pediatric acute myeloid leukemia (Tao et al., 2014), bladder (Garcia-Baquero et al., 2014) and adult brain tumors (Hill et al., 2011) as well as hepatocellular carcinoma (Revill et al., 2013), which may lead to proliferative advantages and aggressive phenotypes during tumorigenesis (Suva et al., 2013).

Previous studies showed that the co-occurrence of gene mutations is frequently observed in two or more genes that tend to have mutations simultaneously in cancer patients (Kang et al., 2008; Zhang et al., 2017). Genes that have mutually exclusive mutations are generally involved in the same biological process (Szczurek and Beerenwinkel, 2014). Genomic alterations targeting similar biological processes could be mutually redundant, with one alteration being able to disrupt the affected process, thus identifying mutual exclusive events may facilitate discovering unknown functional interactions (Zhang et al., 2017). Detecting such patterns is crucial for identifying related novel cancerous pathways and potential treatment targets (Szczurek and Beerenwinkel, 2014). However, to date, co-occurrence (CO) and mutually exclusivity (ME) of DNA methylation in human cancers are less explored. Co-methylation has been reported as a new indicator for discovering functional associations between gene pairs in breast cancer (Akulenko and Helms, 2013). Recently, a number of algorithms have been developed for estimating the significance of ME and CO patterns between two genes (Canisius et al., 2016; Hua et al., 2016; Kim et al., 2017). Some of those tools can be used on DNA methylation data (Canisius et al., 2016), making it possible to comprehensively investigate the CO and ME events of DNAm in diverse The Cancer Genome Atlas (TCGA) cancers.

In this study, we first detected the CO and ME events of DNAm in 14 distinct cancers and explored the relationship between related gene pairs at gene expression and DNA methylation level. Then, we constructed a pan-cancer network with filtered Co-occurrence and mutual exclusivity (COME) events and identified functional epigenetic modules consisting of genes in the zinc finger family. We also found that the selected CO and ME events could be used to classify different types of tumors including pan-cancer into several subtypes with significantly different progression-free interval (PFI). Interestingly, the subtypes determined by COME events are significantly correlated with distinct clinical features, including age, gender, histological type, neoplasm histologic grade, and pathologic stage. Our results suggest that the COME events of DNA methylation could play important roles in tumorigenesis and may benefit the prognosis of different cancers.

## Materials and Methods

### Data Source

The gene expression and DNA methylation data of the TCGA project were downloaded from UCSC Xena^1^ and preprocessed as we previously described (Ding et al., 2019; Ji et al., 2019). The clinical data matrix of TCGA cancers was downloaded from the TCGA Pan-Cancer Clinical Data Resource (TCGA-CDR) (Liu et al., 2018). The statistics of clinical information in 14 cancer types are listed in Supplementary Table S1.

### Definition of Methylated and Unmethylated Genes

We first assigned the DNAm values for each gene with the average beta value of the probes mapped to promoter region, including TSS1500 (from −1,500 to −200 bp upstream of the TSS), TSS200 (region from −200 bp upstream to the transcription start site (TSS) itself), 1stExon (the first exon), and 5’UTR in order as previously described (Jiao et al., 2014; Sharma et al., 2016). According to previous studies (Sproul et al., 2011, 2012; Heyn et al., 2016), a beta value threshold of 0.3 was used to separate methylated from unmethylated probes. In this study, we defined methylated (average CpG DNAm beta values within gene promoter >0.3) and unmethylated (average CpG DNAm beta values within gene promoter <0.3) genes at the threshold of 0.3 in the lack of a better way to dichotomize continuous DNA methylation beta values.

### Cancer Genes and Tumor Suppressor Genes

Cancer genes (CGs) were obtained from the database of CCGD (Abbott et al., 2015), DriverDB (Cheng et al., 2014) and CGC (v84) (Futreal et al., 2004), and tumor suppressor genes (TPG) were downloaded from TSGene (Zhao et al., 2016) database.

### Identification of Co-occurrence and Mutual Exclusive Gene Pairs

We first convert the DNA methylation profile to a binary matrix, in which methylated genes were set to 1 in corresponding patients, and unmethylated genes were set to 0. Then we used DISCOVER (Canisius et al., 2016), a novel statistical independence test that assesses both COME gene pairs by counting how many samples have an alteration in both genes and comparing this to the number of samples expected to have such an overlap by chance if these alterations were independent. DISCOVER algorithm accepts a binary matrix that each row represents a gene and each column represents a patient as an input, then output the result of significant CO or ME gene pairs.

### Filtration of COME Events

Firstly, Fisher’s exact test was performed, for each COME event E*_*i*_*, a contingency table (a, b, c, d) was created as bellow:

**Table:** 

	**Occurred**	**Not Occurred**
Tumor	a	b
Normal	c	d

In the table, *a* and *b* denote the number of tumor samples in which event E*_*i*_* occurred and not occurred, respectively, whereas *c* and *d* separately represent the number of normal samples in which event E*_*i*_* occurred and not occurred respectively. Then Fisher’s exact test (SciPy package in Python) *p*-value was calculated to evaluate whether E*_*i*_* was significantly differentially occurred in this cancer type. Finally, frequently occurred COME events were defined as the events that were significantly differentially occurred in at least three different cancer types.

### Construction of FEM Models

The FEM algorithm (Jiao et al., 2014) is a functional supervised algorithm, which uses a network of relations between genes (in our case, is frequently occurred COME network) to identify subnetworks where a significant number of genes are associated with a phenotype of interest (POI, in our case, is the differential expression and differential methylation). Differential expression and differential methylation analysis were implemented inside the FEM algorithm.

### Unsupervised Consensus Clustering and Survival Analysis

K-means clustering in R package ConsensusClusterPlus (Wilkerson and Hayes, 2010) was used to perform consensus clustering. The optimal cluster number k was chosen depending on the elbow and CDF curve (Senbabaoglu et al., 2014). For survival analysis of the pan-cancer, the best cluster number was chosen as the one with the maximum average silhouette coefficient. Python package lifelines^2^ was implemented in survival analysis, and the log-rank test was used to estimate the significance of different groups.

### Gene Ontology and KEGG Pathway Enrichment Analysis

Gene Ontology (GO) biological process and KEGG pathway enrichment analysis were performed using the web-based gene annotation tools DAVID (Huang da et al., 2009a, b) and ToppGene (Chen et al., 2009), the terms with FDR ≤ = 0.05 were considered as significant.

### Statistical Analysis

All statistical analyses were performed with Python3.5.2 on anaconda3-4.0.0. Kruskal-Wallis *H*-test and Chi-square test were performed with Python package SciPy (Jones et al., 2014).

## Results

### Overview of Co-occurrence and Mutual Exclusivity Network of DNA Methylation in Different Cancers

**FIGURE 1:**
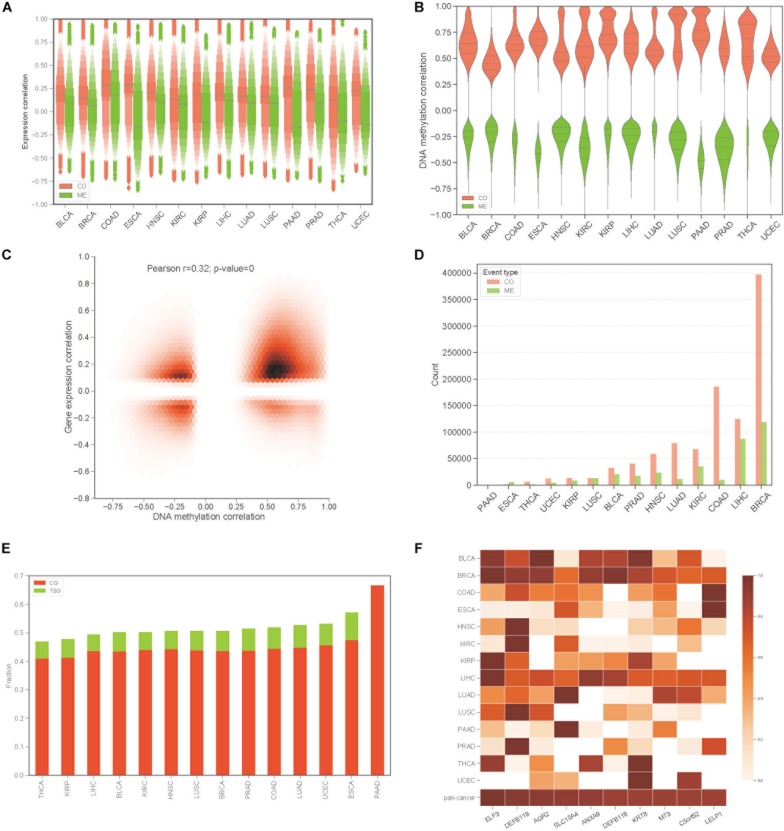
Identification of co-occurrence and mutual exclusivity events of methylation. **(A)** Letter value (LV) plot of Pearson’s correlation coefficient of gene expression level between gene pairs for CO and ME events. **(B)** Pearson’s correlation plot of DNA methylation between gene pairs. **(C)** Jointplot of Pearson’s correlation coefficient between gene expression and DNA methylation for COME gene pairs. **(D)** Countplot of COME events in 14 cancers. **(E)** The proportion of cancer genes and tumor suppressor genes against all of the genes involving in COME events in different cancers. **(F)** Degree distribution of top 10 genes in pan-cancer network for each cancer and pan-cancer.

To construct the COME network of DNA methylation in cancers, we first dichotomized the DNA methylation beta values in every sample with threshold of 0.3, the genes with average beta value ≥ 0.3 in promoter region are designated as methylated while the genes with average beta value lower than 0.3 in promoter regions were considered as unmethylated (see section “Materials and Methods”). Thus, a binary matrix was built for each of 14 different cancers, in which 1 represents methylated and 0 for unmethylated. Then DISCOVER algorithm (Canisius et al., 2016) was employed to detect the CO and ME events based on the binary alteration matrix of DNA methylation. A total of 2,670,651 CO and 2,457,681 ME events that were identified as significant by DISCOVER in 14 cancers (*q*-value ≤ 0.05, *q*-value was calculated by DISCOVER). The expression correlation between genes pair of CO is significantly higher than those of ME (Figure 1A, *p*-value <0.001, independent Student’s *t*-test). Moreover, gene pairs of CO events were mainly positively correlated at the DNA methylation level, whereas gene pairs of ME events were negatively correlated (Figure 1B). In addition, co-methylated gene pairs tend to co-expressed (Figure 1C, Pearson’s correlation = 0.32, *p*-value = 0).

Then, to screen COME events that are associated with the tumor, Fisher exact test was performed in each cancer to screen the COME events that were significantly enriched in tumor or normal samples (*p*-value ≤ 0.05, see section “Materials and Methods”). After filtration, a total of 1,385,366 COME events (involving 7334 unique genes) were retained for further analyses [including 1,029,686 CO (involving 6894 genes) and 355,680 ME events (involving 6924 genes), the distribution of COME events in 14 cancers is shown in Figure 1D]. To explore the associations between COME events and cancers, we calculated the fraction of tumor suppressor genes (TSGs) and disease genes against all genes set involved in COME events. Strikingly, over 45% of the COME genes are cancer genes or TSGs (Figure 1E). We further constructed pan-cancer networks based on CO and ME events. Interestingly, 5 out of the top 10 hub genes in the network are cancer genes, including ELF3, SLC10A4, ANXA9, DEFB118, KRT8 (Figure 1F). Specifically, ELF3 was annotated as a cancer gene for rectum adenocarcinoma, colorectal neoplasms, hematologic diseases and breast neoplasms in the database of DriverDB (Cheng et al., 2014), CoReCG (Agarwal et al., 2016), and DDMGD (Bin Raies et al., 2015) respectively. Moreover, aberrant methylation of hub gene AGR2 was reported to be associated with ovarian cancer (Sung et al., 2014), while MT3 was a putative tumor suppressor gene in pediatric acute myeloid leukemia (Tao et al., 2014).

### Gene Pairs of COME Events Tend to Be Significantly Correlated Between DNA Methylation and Gene Expression

**FIGURE 2:**
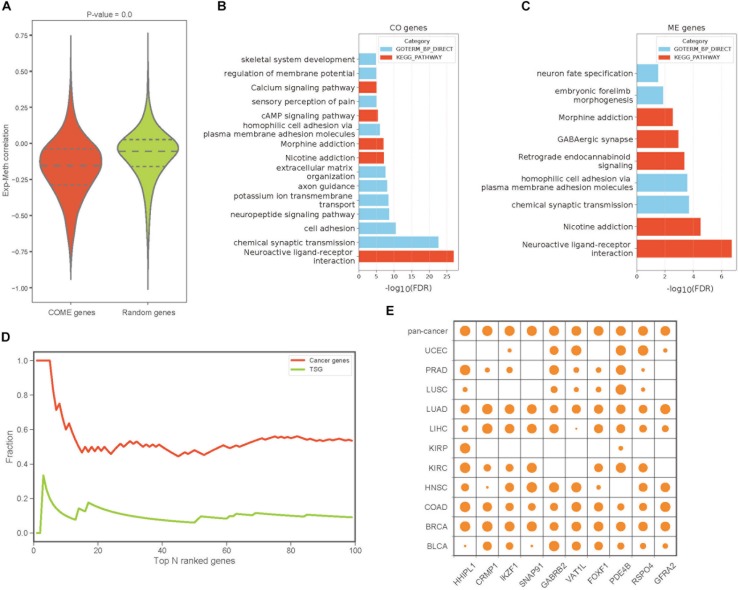
The features and functions of gene pairs in COME events. **(A)** Box plot of Pearson’s correlation coefficient between gene expression and DNA methylation for the genes involved in COME events compared with that of random genes set (*p*-value was calculated by independent Student’s *t*-test). **(B**,**C)** Enriched KEGG pathways and biological processes for the genes involved in CO and ME events (DAVID online web server). **(D)** Fraction of cancer genes and tumor suppressor genes (TSG) against the top N genes ranked by the degree in pan-cancer network. **(E)** Degree distribution of top 10 genes in pan-cancer network for each cancer and pan-cancer.

To build a reliable network of COME events in each cancer, we further screened frequently occurred events from the above 1,385,366 COME events that were significantly enriched in tumor or normal samples, as frequently occurred events, we considered events that were differentially occurred in at least three different cancer types. After filtering, we found that gene expression correlation and DNA methylation correlation between gene pairs of COME events tend to be more correlated than that of before-filtering (Pearson’s correlation *r* = 0.55, *p*-value = 0, Supplementary Figure S1A). The correlations between gene expression and DNA methylation of most genes involved in COME gene pairs tend to be more negatively correlated than that of randomly generated random gene sets (Figure 2A, random gene set was generated randomly with the same number of genes in each cancer). Gene functional enrichment analysis showed that the genes involved in CO events were mainly enriched in the pathways of Neuroactive ligand-receptor interaction, Nicotine addiction, Morphine addiction, cAMP signaling, Calcium signaling and the biological processes of chemical synaptic transmission, cell adhesion, neuropeptide signaling pathway and so on (Figure 2B and Supplementary Table S2). While the genes of ME events were mainly associated with the development of the central nervous system and brain (Figure 2C and Supplementary Table S2). We further built a pan-cancer cooperative network by merging the networks of each cancer (Supplementary Figures S1B–D). Intriguingly, most of the genes with a high degree (have the largest number of links) in the pan-cancer network showed enrichment for known cancer-related genes or tumor suppressor genes (Figure 2D). Aberrant DNA methylation of some of those top 10 genes with the highest degree has been reported to be associated with neoplasms, such as HHIPL1 (Duong et al., 2012), GABRB2 (Beltrami et al., 2017), FOXF1 (Lo et al., 2010) and RSPO4 (Oka et al., 2009: Figure 2E).

### Zinc Fingers Gene Family Is Enriched in Functional Epigenetic Modules

**FIGURE 3:**
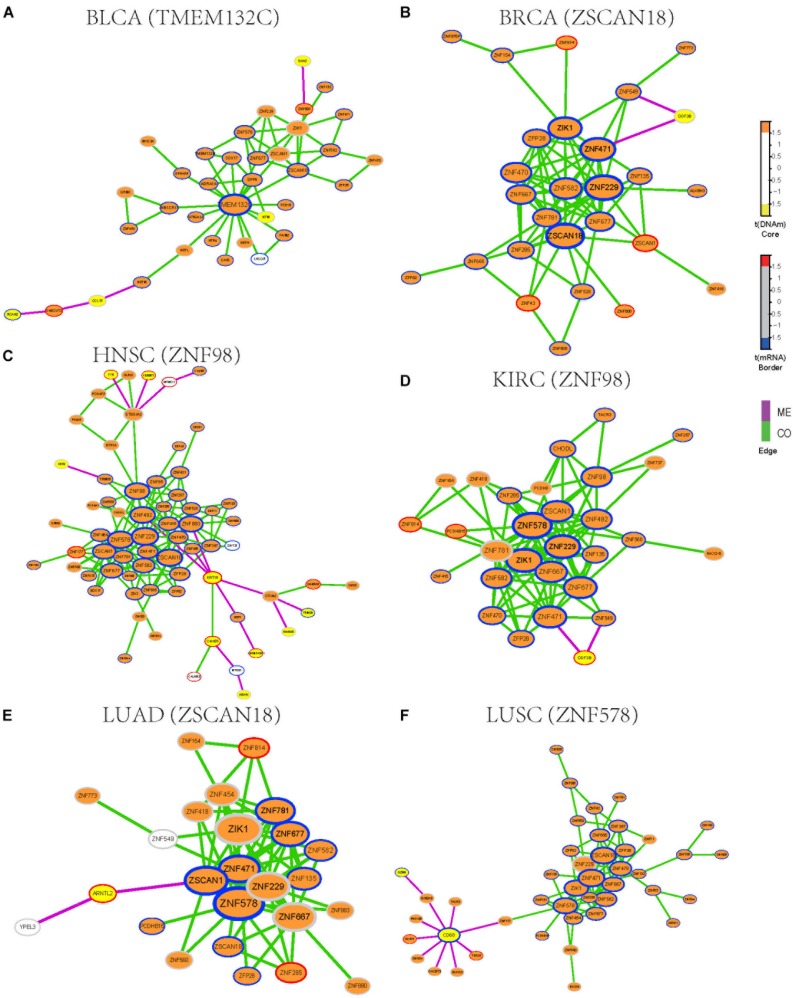
Functional epigenetic modules identified from CO and ME network. The color of core indicate significant DNA methylation changes, color of border represent significant gene expression changes, edges represent COME events between two genes, and edge color indicates different event type (co-occurrence or mutual exclusivity). **(A–F)** Functional epigenetic modules identified from corresponding cancer type.

To identify functional epigenetic modules, we integrated the gene expression and DNA methylation data from TCGA and frequently occurred COME networks constructed in each cancer using the FEM algorithm (Jiao et al., 2014), which can be used to effectively identify gene modules of coordinated differential methylation and differential expression in the context of a network. Many functional epigenetic modules were identified by FEM. Remarkably, six modules enriched in the zinc fingers gene family were identified in 6 distinct cancer types (Figures 3A–F). Most genes in these modules were hypermethylated and down-regulated, indicating that genes of zinc fingers family may tend to be co-methylated and transcriptionally suppressed.

To further explore the associations between aberrant DNA methylation of zinc fingers gene family and neoplasms, we found that those genes were significantly enriched in regulation of transcription, DNA binding transcription factor activity, RNA polymerase II regulatory region sequence-specific DNA binding, Neuroactive ligand-receptor interaction, and so on (Supplementary Table S3). Besides, many of the genes in these modules were enriched in cytoband of 19q13.43, transcription factor binding sites of ZNF274 and they tend to have similar DNA methylation patterns.

We also examined whether those genes in the zinc fingers gene family can be used to distinguish tumor samples from normal samples in the above 14 cancers. Six genes (ZIK1, ZNF471, ZNF229, ZFP28, ZNF677, and ZNF582) shared among 6 modules were selected to build a logistic regression module. Compared with the models based on gene expression or DNA methylation along (AUC = 0.76), the module integrated both DNA methylation and gene expression data of the 6 genes showed better performance in distinguishing tumor samples from normal (AUC = 0.86, Supplementary Figure S2). Moreover, the clustering result also indicates that gene expression and DNA methylation profile of those 6 genes can effectively separate tumor samples from normal samples (Supplementary Figure S3). Most of those 6 genes are involved in function of nucleic acid binding, and some of those genes have been reported to be aberrantly methylated in tumors [such as ZIK1 (Borinstein et al., 2010), ZNF471 (Bhat et al., 2017)] and may serve as a marker of cancer [e.g., ZNF677 (Heller et al., 2015), ZNF582 (Lin et al., 2014)]. Consequently, our finding demonstrates that combing DNA methylation and gene expression data of those genes from zinc fingers family may be associated with tumorigenesis.

### Co-occurrence and Mutual Exclusive Events Contribute to Prognosis in Human Cancers

**FIGURE 4:**
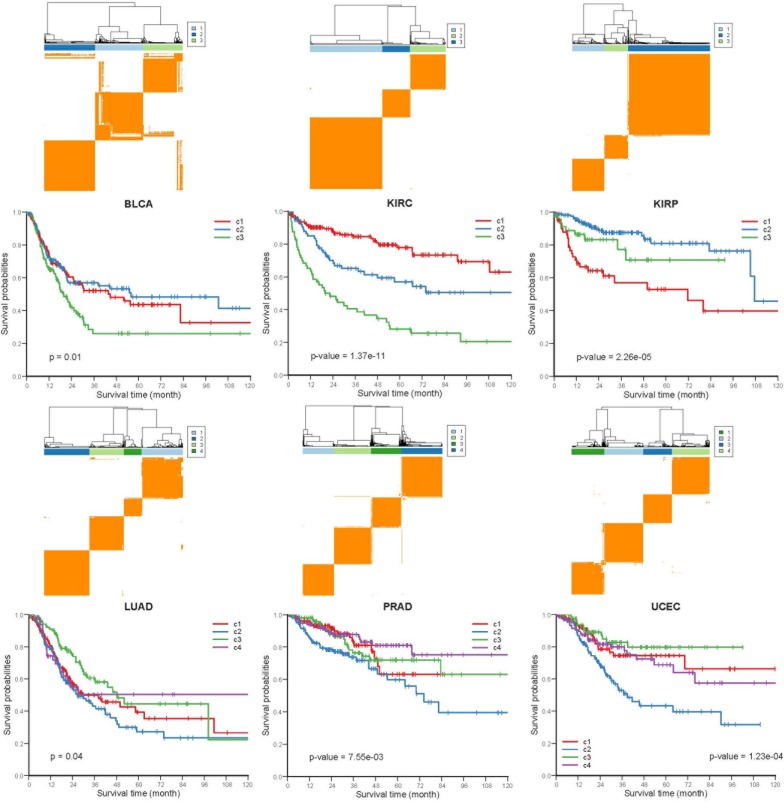
Kaplan-Meier plot of PFI for distinct subtypes of 6 different cancers. Consensus cluster plot (top) and Kaplan-Meier survival plots (bottom) were separately shown for 6 disparate cancers, c1-c4 represent cluster 1 – cluster 4, and *p*-values were calculated by log rank test.

To investigate whether the COME events are associated with cancer prognosis, Frequently occurred COME events (occurred in at least 3 distinct cancer types, Supplementary Table S4) were used to perform consensus cluster and Kaplan-Meier survival analysis in each of those 13 cancers (no frequently occurred COME event was left in pancreatic adenocarcinoma, PAAD). Strikingly, we observed significantly different PFI among disparate subtypes in 6 different cancer types (*p*-value <0.05, log-rank test, Figure 4), as well as in BRCA and lung squamous cell carcinoma (LUSC) (*p*-value = 0.07, Supplementary Figure S4).

**FIGURE 5:**
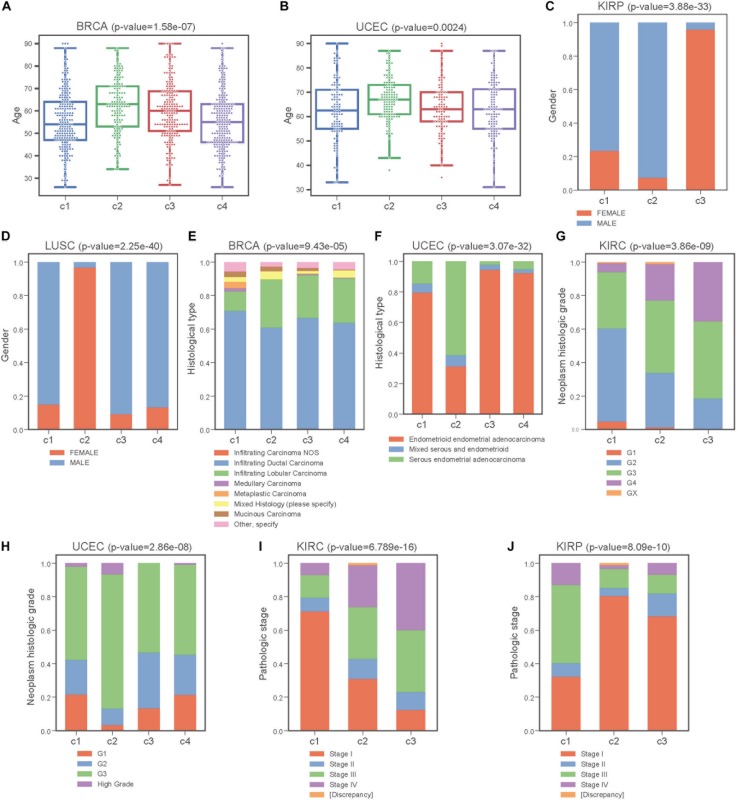
COME subtypes correlate with distinct clinical features. **(A,B)** Age distribution of different COME subtypes identified in BRCA and UCEC, respectively. **(C–J)** Distribution of gender, histological type, neoplasm histologic grade and pathologic stage against COME subtypes in corresponding TCGA cancers. *p*-values for continuous variable were calculated by using Kruskal-Wallis *H*-test **(A,B)**, and *p*-values for categorical variable were calculated by performing Chi-square test **(C–J)**. *p*-value were calculated between all different groups.

Next, we performed Kruskal-Wallis *H*-test and Chi-square test to explore the association between COME subtypes and different clinical information (including age, gender, histological type, neoplasm histologic grade, and pathologic stage). Interestingly, significant difference was observed between different clinical features and different subtypes in 5 cancers (Figure 5). For example, the distribution of age was significantly different in different COME subtypes in BRCA and uterine corpus endometrial carcinoma (UCEC) (Figures 5A,B, Kruskal-Wallis *H*-test, *p*-value <0.001), which is in accordance with previous studies that DNA methylation is associated with age (Horvath, 2013; Johnson et al., 2014). In kidney renal papillary cell carcinoma (KIRP), cluster 3 is enriched with female whereas cluster 2 is enriched with male (Figure 5C). Moreover, cluster 2 of LUSC is significantly enriched with female, which is opposite to clusters 1, 3, and 4 (Figure 5D). We also found that different COME subtypes have distinct distribution of histological types in BRCA and UCEC (Figures 5E,F). Furthermore, cluster 2 of UCEC is enriched with the histological type of serous endometrial adenocarcinoma and cluster 2 has a shorter PFI compared to other clusters (Figure 4). As for neoplasm histologic grade (Figures 5G,H), cluster 3 of kidney renal clear cell carcinoma (KIRC) is enriched in grade G3 and G4, and poorer prognosis was observed in this cluster (Figure 4). Similarly, G3 and high-grade patients are enriched in cluster 2 of UCEC, while cluster 2 has poorer survival probability compared to other subtypes (Figure 4). Patients of stage III and stage IV are enriched in cluster 3 and has the poorest clinical outcome in KIRC, while cluster 1 is enriched with the patients of stage III and stage IV and has the shortest PFI in KIRP (Figures 4, 5I,J). Collectively, the subtypes determined by COME pattern are correlated with various clinical features (age, gender, histological type, neoplasm histologic grade, and pathologic stage), which may explain why distinct COME subtypes have significantly different PFI.

**FIGURE 6:**
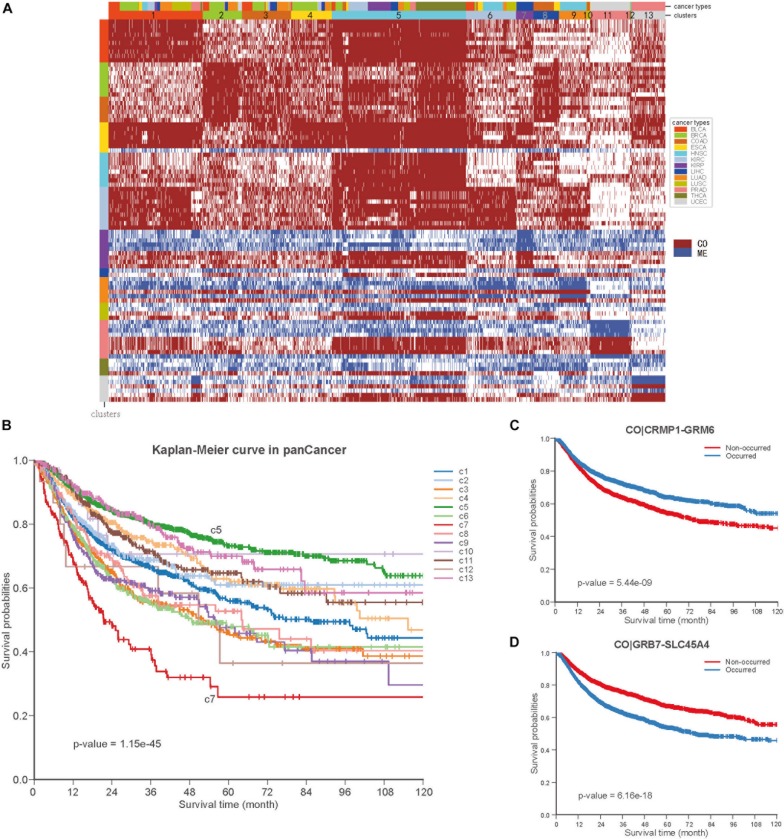
Co-occurrence and mutual exclusive events contribute to prognosis of pan-cancer. **(A)** Clustering heatmap based on top 15 enriched COME events in 13 clusters. The clusters are denoted by number and color in the second bar, and tissue of origin specified in the first color bar. The color in the left identified top 15 COME pairs significantly enriched in corresponding cluster. **(B)** Kaplan-Meier plot for the PFI of 13 clusters identified in pan-cancer. **(C,D)** Kaplan-Meier plot of PFI for co-methylation event of CRMP1-GRM6 and GRB7-SLC45A4 in pan-cancer, respect.

To further explore the COME events in pan-cancer, we performed consensus clustering based on the frequently occurred COME events in 5442 tumor samples of 13 types of cancer. Thirteen clusters were identified by maximizing the average silhouette coefficient. The top 15 significantly enriched COME events in each cluster are shown in Figure 6A. Most of these clusters were significantly correlated with cancer tissue of origin (*p*-value <0.0001, Chi-square test, Supplementary Table S5). For example, clusters of C7, C9, C10, C11, and C13 were significantly enriched with patients from LIHC (liver hepatocellular carcinoma), HNSC (head and neck squamous cell carcinoma), COAD (colon adenocarcinoma), UCEC and PRAD (prostate adenocarcinoma), respectively (*p*-value <0.0001, hypergeometric test). While some other clusters, such as C1, contained a mixture type of BLCA (bladder urothelial carcinoma), BRCA, LUAD (lung adenocarcinoma), and LUSC (*p*-value <0.0001, hypergeometric test). Furthermore, those 13 clusters exhibited significantly different PFI via Kaplan-Meier analysis (*p*-value <0.0001, log-rank test, Figure 6B). Among the 13 clusters, cluster C5 exhibited the best prognosis and the co-occurrence of CRMP1-GRM6 was the most significantly enriched event in this cluster (*p*-value <0.001, hypergeometric test). CRMP1 (collapsin response mediator protein 1) has been reported to be associated with medulloblastoma (Li et al., 2015) and gliomas (Mukherjee et al., 2009). Hypermethylation of the CpG sites on GRM6 (glutamate metabotropic receptor 6) was reported to be a hallmark of CIMP in clear cell renal cell carcinomas (Arai et al., 2012). In contrast, cluster C7 was associated with the poorest prognosis and co-methylation of GRB7-SLC45A4 was enriched in this group (*p*-value <0.001, hypergeometric test). GRB7 (growth factor receptor bound protein 7) was reported to play an important role in breast cancer progression (Lim et al., 2014). We further performed Kaplan-Meier survival analysis in the pan-cancer to verify whether the co-methylation of CRMP1-GRM6 and GRB7-SLC45A4 was associated with clinical outcome in pan-cancer. The result shows that patients with co-methylation of CRMP1-GRM6 have better outcomes (*p*-value <0.0001, log-rank test, Figure 6C), whereas patients with co-methylation of GRB7-SLC45A4 exhibit significantly poorer prognosis (*p*-value <0.0001, log-rank test, Figure 6D).

## DISCUSSION

In our study, we first identified 2,670,651 CO and 2,457,681 ME gene pairs in 14 different cancers based on the methylation profile from the TCGA project. Interestingly, the genes in functional epigenetic modules identified in six cancer types were mainly from the zinc finger gene family, and most of those genes were epigenetically repressed. Although several studies have reported the epigenetic silencing of the zinc finger gene family (Lleras et al., 2011; Severson et al., 2013; Gaykalova et al., 2015), we are the first to identify functional epigenetic modules of the zinc finger gene family in six cancer types by integrating gene expression and DNA methylation data in the context of COME networks. Methylation was reported to be the main mechanism for downregulation of tumor cell growth suppressor ZNF677 in non-small cell lung cancers (NSCLCs) and the methylation of ZNF677 could be used in the prognosis of NSCLCs (Heller et al., 2015). Furthermore, we identified a set of COME events that can divide tumor patients into different subtypes with significantly different clinical outcomes. Different COME subtypes were found to be significantly associated with distinct clinical features, such as age, gender, histological type, neoplasm histologic grade and pathologic stage. We also found that COME events could be used to divide tumor samples of pan-cancer into different subtypes with significantly different outcomes, which may benefit the prediction of the prognosis for pan-cancer.

This study is just the beginning to investigate and characterize the roles of COME of DNA methylation in human cancers. Our findings may contribute to the diagnosis and prognosis of human pan-cancer. The underlying mechanism and function of COME events in diverse cancers are still needed to be further studied in the future.

## Data Availability Statement

The TCGA data analyzed in this study was obtained from UCSC Xena (http://xena.ucsc.edu). This study fully complies with the TCGA publication requirements (http://cancergenome.nih.gov/publications/publicationguidelines).

## Author Contributions

TS and WD designed the study and wrote the manuscript. WD and YH conducted the data analysis. WD, GC, and TS revised and finalized the manuscript. All authors read and approved the final manuscript.

## Conflict of Interest

The authors declare that the research was conducted in the absence of any commercial or financial relationships that could be construed as a potential conflict of interest.

## Footnotes

1 http://xena.ucsc.edu

2 http://lifelines.readthedocs.io/en/latest/index.html

## Abbreviations

BLCA: Bladder Urothelial Carcinoma
BRCA: Breast invasive carcinoma
COAD: Colon adenocarcinoma
ESCA: Esophageal carcinoma
HNSC: Head and Neck squamous cell carcinoma
KIRC: Kidney renal clear cell carcinoma
KIRP: Kidney renal papillary cell carcinoma
LIHC: Liver hepatocellular carcinoma
LUAD: Lung adenocarcinoma
LUSC: Lung squamous cell carcinoma
PAAD: Pancreatic adenocarcinoma
PRAD: Prostate adenocarcinoma
THCA: Thyroid carcinoma
UCEC: Uterine Corpus Endometrial Carcinoma.

## References

[B1] Abbott K. L. Nyre E. T. Abrahante J. Ho Y. Y. Isaksson Vogel R. Starr T. K. (2015). The candidate cancer gene database: a database of cancer driver genes from forward genetic screens in mice. *Nucleic Acids Res.* 43 D844–D848. 10.1093/nar/gku770 25190456 PMC4384000

[B2] Agarwal R. Kumar B. Jayadev M. Raghav D. Singh A. (2016). CoReCG: a comprehensive database of genes associated with colon-rectal cancer. *Database* 2016:baw059. 10.1093/database/baw059 27114494 PMC4843536

[B3] Akulenko R. Helms V. (2013). DNA co-methylation analysis suggests novel functional associations between gene pairs in breast cancer samples. *Hum. Mol. Genet.* 22 3016–3022. 10.1093/hmg/ddt158 23571108

[B4] Arai E. Chiku S. Mori T. Gotoh M. Nakagawa T. Fujimoto H. (2012). Single-CpG-resolution methylome analysis identifies clinicopathologically aggressive CpG island methylator phenotype clear cell renal cell carcinomas. *Carcinogenesis* 33 1487–1493. 10.1093/carcin/bgs177 22610075 PMC3418891

[B5] Beltrami C. M. Dos Reis M. B. Barros-Filho M. C. Marchi F. A. Kuasne H. Pinto C. A. L. (2017). Integrated data analysis reveals potential drivers and pathways disrupted by DNA methylation in papillary thyroid carcinomas. *Clin. Epigenet.* 9:45. 10.1186/s13148-017-0346-2 28469731 PMC5414166

[B6] Bhat S. Kabekkodu S. P. Jayaprakash C. Radhakrishnan R. Ray S. Satyamoorthy K. (2017). Gene promoter-associated CpG island hypermethylation in squamous cell carcinoma of the tongue. *Virchows Arch.* 470 445–454. 10.1007/s00428-017-2094-2 28255813

[B7] Bin Raies A. Mansour H. Incitti R. Bajic V. B. (2015). DDMGD: the database of text-mined associations between genes methylated in diseases from different species. *Nucleic Acids Res.* 43 D879–D886. 10.1093/nar/gku1168 25398897 PMC4383966

[B8] Borinstein S. C. Conerly M. Dzieciatkowski S. Biswas S. Washington M. K. Trobridge P. (2010). Aberrant DNA methylation occurs in colon neoplasms arising in the azoxymethane colon cancer model. *Mol. Carcinog.* 49 94–103. 10.1002/mc.20581 19777566 PMC2875385

[B9] Canisius S. Martens J. W. Wessels L. F. (2016). A novel independence test for somatic alterations in cancer shows that biology drives mutual exclusivity but chance explains most co-occurrence. *Genome Biol.* 17:261. 10.1186/s13059-016-1114-x 27986087 PMC5162102

[B10] Chen J. Bardes E. E. Aronow B. J. Jegga A. G. (2009). ToppGene Suite for gene list enrichment analysis and candidate gene prioritization. *Nucleic Acids Res.* 37 W305–W311. 10.1093/nar/gkp427 19465376 PMC2703978

[B11] Cheng W. C. Chung I. F. Chen C. Y. Sun H. J. Fen J. J. Tang W. C. (2014). DriverDB: an exome sequencing database for cancer driver gene identification. *Nucleic Acids Res.* 42 D1048–D1054. 10.1093/nar/gkt1025 24214964 PMC3965046

[B12] Delpu Y. Cordelier P. Cho W. C. Torrisani J. (2013). DNA methylation and cancer diagnosis. *Int. J. Mol. Sci.* 14 15029–15058. 10.3390/ijms140715029 23873296 PMC3742286

[B13] Ding W. Chen G. Shi T. (2019). Integrative analysis identifies potential DNA methylation biomarkers for pan-cancer diagnosis and prognosis. *Epigenetics* 14 67–80. 10.1080/15592294.2019.1568178 30696380 PMC6380428

[B14] Duong C. V. Emes R. D. Wessely F. Yacqub-Usman K. Clayton R. N. Farrell W. E. (2012). Quantitative, genome-wide analysis of the DNA methylome in sporadic pituitary adenomas. *Endocr. Relat. Cancer* 19 805–816. 10.1530/ERC-12-0251 23045325

[B15] Futreal P. A. Coin L. Marshall M. Down T. Hubbard T. Wooster R. (2004). A census of human cancer genes. *Nat. Rev. Cancer* 4 177–183. 10.1038/nrc1299 14993899 PMC2665285

[B16] Garcia-Baquero R. Puerta P. Beltran M. Alvarez-Mujica M. Alvarez-Ossorio J. L. Sanchez-Carbayo M. (2014). Methylation of tumor suppressor genes in a novel panel predicts clinical outcome in paraffin-embedded bladder tumors. *Tumour Biol.* 35 5777–5786. 10.1007/s13277-014-1767-6 24577895

[B17] Gaykalova D. A. Vatapalli R. Wei Y. Tsai H. L. Wang H. Zhang C. (2015). Outlier analysis defines zinc finger gene family DNA methylation in tumors and saliva of head and neck cancer patients. *PLoS One* 10:e0142148. 10.1371/journal.pone.0142148 26544568 PMC4636259

[B18] Heller G. Altenberger C. Schmid B. Marhold M. Tomasich E. Ziegler B. (2015). DNA methylation transcriptionally regulates the putative tumor cell growth suppressor ZNF677 in non-small cell lung cancers. *Oncotarget* 6 394–408. 10.18632/oncotarget.2697 25504438 PMC4381603

[B19] Heyn H. Vidal E. Ferreira H. J. Vizoso M. Sayols S. Gomez A. (2016). Epigenomic analysis detects aberrant super-enhancer DNA methylation in human cancer. *Genome Biol.* 17:11. 10.1186/s13059-016-0879-2 26813288 PMC4728783

[B20] Hill V. K. Underhill-Day N. Krex D. Robel K. Sangan C. B. Summersgill H. R. (2011). Epigenetic inactivation of the RASSF10 candidate tumor suppressor gene is a frequent and an early event in gliomagenesis. *Oncogene* 30 978–989. 10.1038/onc.2010.471 20956940

[B21] Horvath S. (2013). DNA methylation age of human tissues and cell types. *Genome Biol.* 14:R115. 10.1186/gb-2013-14-10-r115 24138928 PMC4015143

[B22] Hua X. Hyland P. L. Huang J. Song L. Zhu B. Caporaso N. E. (2016). MEGSA: a powerful and flexible framework for analyzing mutual exclusivity of tumor mutations. *Am. J. Hum. Genet.* 98 442–455. 10.1016/j.ajhg.2015.12.021 26899600 PMC4800034

[B23] Huang da W. Sherman B. T. Lempicki R. A. (2009a). Bioinformatics enrichment tools: paths toward the comprehensive functional analysis of large gene lists. *Nucleic Acids Res.* 37 1–13. 10.1093/nar/gkn923 19033363 PMC2615629

[B24] Huang da W. Sherman B. T. Lempicki R. A. (2009b). Systematic and integrative analysis of large gene lists using DAVID bioinformatics resources. *Nat. Protoc* 4 44–57. 10.1038/nprot.2008.211 19131956

[B25] Ji X. Tong W. Ning B. Mason C. E. Kreil D. P. Labaj P. P. (2019). QuaPra: efficient transcript assembly and quantification using quadratic programming with Apriori algorithm. *Sci. China Life Sci.* 62 937–946. 10.1007/s11427-018-9433-3 31124003

[B26] Jiao Y. Widschwendter M. Teschendorff A. E. (2014). A systems-level integrative framework for genome-wide DNA methylation and gene expression data identifies differential gene expression modules under epigenetic control. *Bioinformatics* 30 2360–2366. 10.1093/bioinformatics/btu316 24794928

[B27] Johnson K. C. Koestler D. C. Cheng C. Christensen B. C. (2014). Age-related DNA methylation in normal breast tissue and its relationship with invasive breast tumor methylation. *Epigenetics* 9 268–275. 10.4161/epi.27015 24196486 PMC3962537

[B28] Jones E. Oliphant T. Peterson P. (2014). *{SciPy}: Open Source scientific Tools for {Python}.*

[B29] Kang S. Seo S. S. Chang H. J. Yoo C. W. Park S. Y. Dong S. M. (2008). Mutual exclusiveness between PIK3CA and KRAS mutations in endometrial carcinoma. *Int. J. Gynecol. Cancer* 18 1339–1343. 10.1111/j.1525-1438.2007.01172.x 18221484

[B30] Kim Y. A. Madan S. Przytycka T. M. (2017). WeSME: uncovering mutual exclusivity of cancer drivers and beyond. *Bioinformatics* 33 814–821. 10.1093/bioinformatics/btw242 27153670 PMC5888950

[B31] Li K. K. Qi Y. Xia T. Yao Y. Zhou L. Lau K. M. (2015). CRMP1 inhibits proliferation of medulloblastoma and is regulated by HMGA1. *PLoS One* 10:e0127910. 10.1371/journal.pone.0127910 26009886 PMC4444180

[B32] Lim R. C. Price J. T. Wilce J. A. (2014). Context-dependent role of Grb7 in HER2+ve and triple-negative breast cancer cell lines. *Breast Cancer Res. Treat.* 143 593–603. 10.1007/s10549-014-2838-5 24464577

[B33] Lin H. Chen T. C. Chang T. C. Cheng Y. M. Chen C. H. Chu T. Y. (2014). Methylated ZNF582 gene as a marker for triage of women with Pap smear reporting low-grade squamous intraepithelial lesions - a Taiwanese Gynecologic Oncology Group (TGOG) study. *Gynecol. Oncol.* 135 64–68. 10.1016/j.ygyno.2014.08.012 25134998

[B34] Liu J. Lichtenberg T. Hoadley K. A. Poisson L. M. Lazar A. J. Cherniack A. D. (2018). An integrated TCGA pan-cancer clinical data resource to drive high-quality survival outcome analytics. *Cell* 173 400–416.e11. 10.1016/j.cell.2018.02.052 29625055 PMC6066282

[B35] Lleras R. A. Adrien L. R. Smith R. V. Brown B. Jivraj N. Keller C. (2011). Hypermethylation of a cluster of Kruppel-type zinc finger protein genes on chromosome 19q13 in oropharyngeal squamous cell carcinoma. *Am. J. Pathol.* 178 1965–1974. 10.1016/j.ajpath.2011.01.049 21514414 PMC3081156

[B36] Lo P. K. Lee J. S. Liang X. Han L. Mori T. Fackler M. J. (2010). Epigenetic inactivation of the potential tumor suppressor gene FOXF1 in breast cancer. *Cancer Res.* 70 6047–6058. 10.1158/0008-5472.CAN-10-1576 20587515 PMC2909657

[B37] Mukherjee J. DeSouza L. V. Micallef J. Karim Z. Croul S. Siu K. W. (2009). Loss of collapsin response mediator Protein1, as detected by iTRAQ analysis, promotes invasion of human gliomas expressing mutant EGFRvIII. *Cancer Res.* 69 8545–8554. 10.1158/0008-5472.CAN-09-1778 19903856

[B38] Oka D. Yamashita S. Tomioka T. Nakanishi Y. Kato H. Kaminishi M. (2009). The presence of aberrant DNA methylation in noncancerous esophageal mucosae in association with smoking history: a target for risk diagnosis and prevention of esophageal cancers. *Cancer* 115 3412–3426. 10.1002/cncr.24394 19472401

[B39] Revill K. Wang T. Lachenmayer A. Kojima K. Harrington A. Li J. (2013). Genome-wide methylation analysis and epigenetic unmasking identify tumor suppressor genes in hepatocellular carcinoma. *Gastroenterology* 145 e1–e25. 10.1053/j.gastro.2013.08.055 24012984 PMC3892430

[B40] Senbabaoglu Y. Michailidis G. Li J. Z. (2014). Critical limitations of consensus clustering in class discovery. *Sci. Rep.* 4:6207. 10.1038/srep06207 25158761 PMC4145288

[B41] Severson P. L. Tokar E. J. Vrba L. Waalkes M. P. Futscher B. W. (2013). Coordinate H3K9 and DNA methylation silencing of ZNFs in toxicant-induced malignant transformation. *Epigenetics* 8 1080–1088. 10.4161/epi.25926 23974009 PMC3891689

[B42] Sharma P. Bhunia S. Poojary S. S. Tekcham D. S. Barbhuiya M. A. Gupta S. (2016). Global methylation profiling to identify epigenetic signature of gallbladder cancer and gallstone disease. *Tumour Biology* 37 14687–14699. 10.1007/s13277-016-5355-9 27623942

[B43] Sproul D. Kitchen R. R. Nestor C. E. Dixon J. M. Sims A. H. Harrison D. J. (2012). Tissue of origin determines cancer-associated CpG island promoter hypermethylation patterns. *Genome Biol.* 13:R84. 10.1186/gb-2012-13-10-r84 PMC3491412 23034185

[B44] Sproul D. Nestor C. Culley J. Dickson J. H. Dixon J. M. Harrison D. J. (2011). Transcriptionally repressed genes become aberrantly methylated and distinguish tumors of different lineages in breast cancer. *Proc. Natl. Acad. Sci. U.S.A* 108 4364–4369. 10.1073/pnas.1013224108 21368160 PMC3060255

[B45] Stefansson O. A. Moran S. Gomez A. Sayols S. Arribas-Jorba C. Sandoval J. (2015). A DNA methylation-based definition of biologically distinct breast cancer subtypes. *Mol. Oncol.* 9 555–568. 10.1016/j.molonc.2014.10.012 25468711 PMC5528700

[B46] Sung H. Y. Choi E. N. Lyu D. Park A. K. Ju W. Ahn J. H. (2014). Aberrant hypomethylation-mediated AGR2 overexpression induces an aggressive phenotype in ovarian cancer cells. *Oncol. Rep.* 32 815–820. 10.3892/or.2014.3243 24920423

[B47] Suva M. L. Riggi N. Bernstein B. E. (2013). Epigenetic reprogramming in cancer. *Science* 339 1567–1570. 10.1126/science.1230184 23539597 PMC3821556

[B48] Szczurek E. Beerenwinkel N. (2014). Modeling mutual exclusivity of cancer mutations. *PLoS Computat. Biol.* 10:e1003503. 10.1371/journal.pcbi.1003503 24675718 PMC3967923

[B49] Tahara T. Arisawa T. (2015). DNA methylation as a molecular biomarker in gastric cancer. *Epigenomics* 7 475–486. 10.2217/epi.15.4 26077432

[B50] Tao Y. F. Xu L. X. Lu J. Cao L. Li Z. H. Hu S. Y. (2014). Metallothionein III (MT3) is a putative tumor suppressor gene that is frequently inactivated in pediatric acute myeloid leukemia by promoter hypermethylation. *J. Transl. Med.* 12:182. 10.1186/1479-5876-12-182 24962166 PMC4082423

[B51] Wilkerson M. D. Hayes D. N. (2010). ConsensusClusterPlus: a class discovery tool with confidence assessments and item tracking. *Bioinformatics* 26 1572–1573. 10.1093/bioinformatics/btq170 20427518 PMC2881355

[B52] Zhang H. Deng Y. Zhang Y. Ping Y. Zhao H. Pang L. (2017). Cooperative genomic alteration network reveals molecular classification across 12 major cancer types. *Nucleic Acids Res.* 45 567–582. 10.1093/nar/gkw1087 27899621 PMC5314758

[B53] Zhao M. Kim P. Mitra R. Zhao J. Zhao Z. (2016). TSGene 2.0: an updated literature-based knowledgebase for tumor suppressor genes. *Nucleic Acids Res.* 44 D1023–D1031. 10.1093/nar/gkv1268 26590405 PMC4702895

