# Supplementary material for: Co-occurrence and Mutual Exclusivity Analysis of DNA Methylation Reveals Distinct Subtypes in Multiple Cancers
Source: Front Cell Dev Biol. 2020 Jan 29;8:20. doi: 10.3389/fcell.2020.00020 (PMC7000380; doi:10.3389/fcell.2020.00020)
Supplement: Supplementary file 1 [file Data_Sheet_1.docx]

Supplementary Material

# Supplementary Figures and Tables

## Supplementary Figures


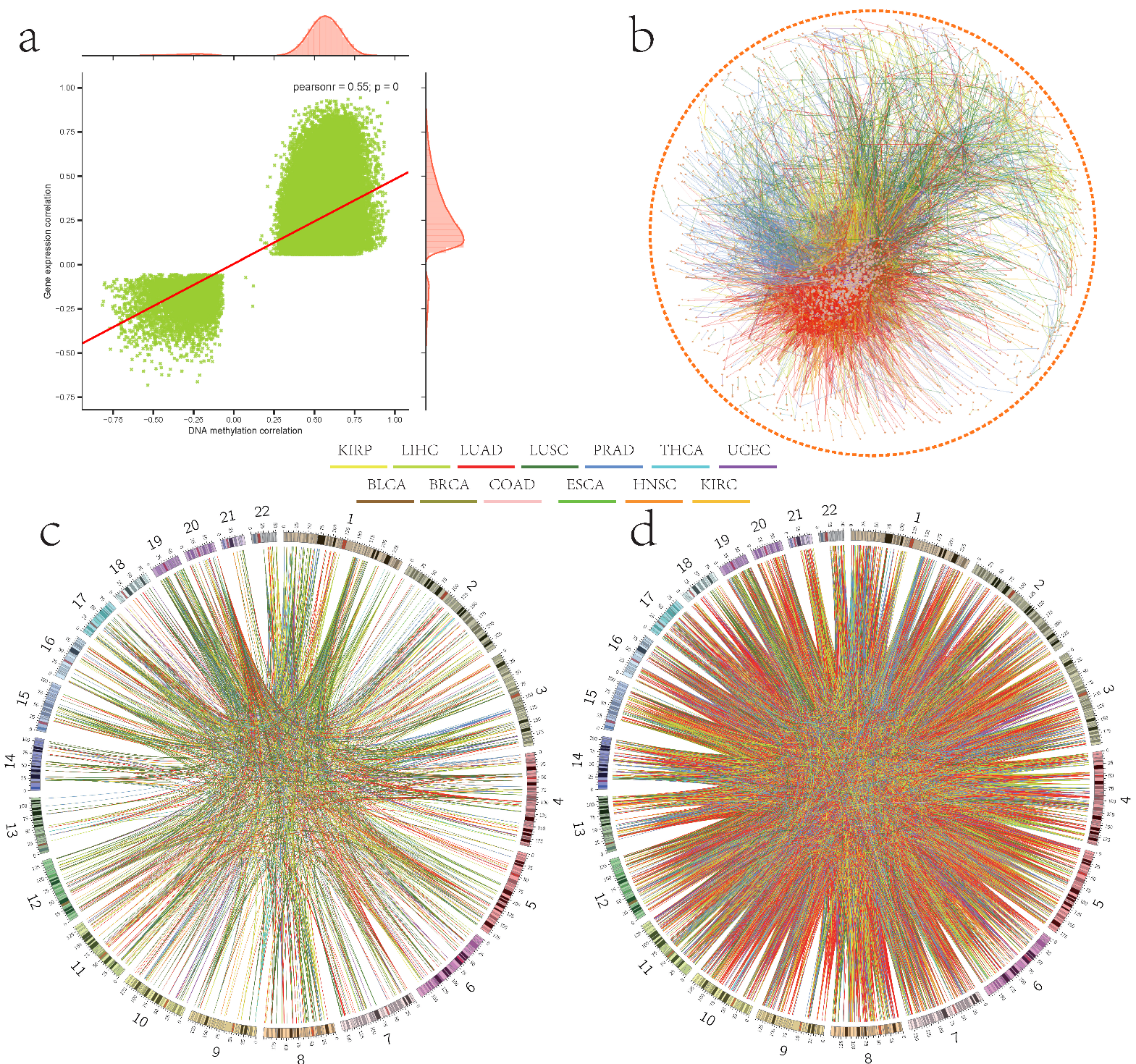


**Supplementary Figure 1.** Construction of frequently occurred CO and ME network. a Jointplot of Pearson’s correlation coefficient between gene expression and DNA methylation of COME gene pairs after filtering. b CO and ME network of pan-cancer merged from 13 cancers. Edges represent CO or ME event between two genes, different colors of edges represent different cancer types. c, d Circos plot for all ME and CO events in pan-cancer, respectively. Different colors of links represent distinct cancers.


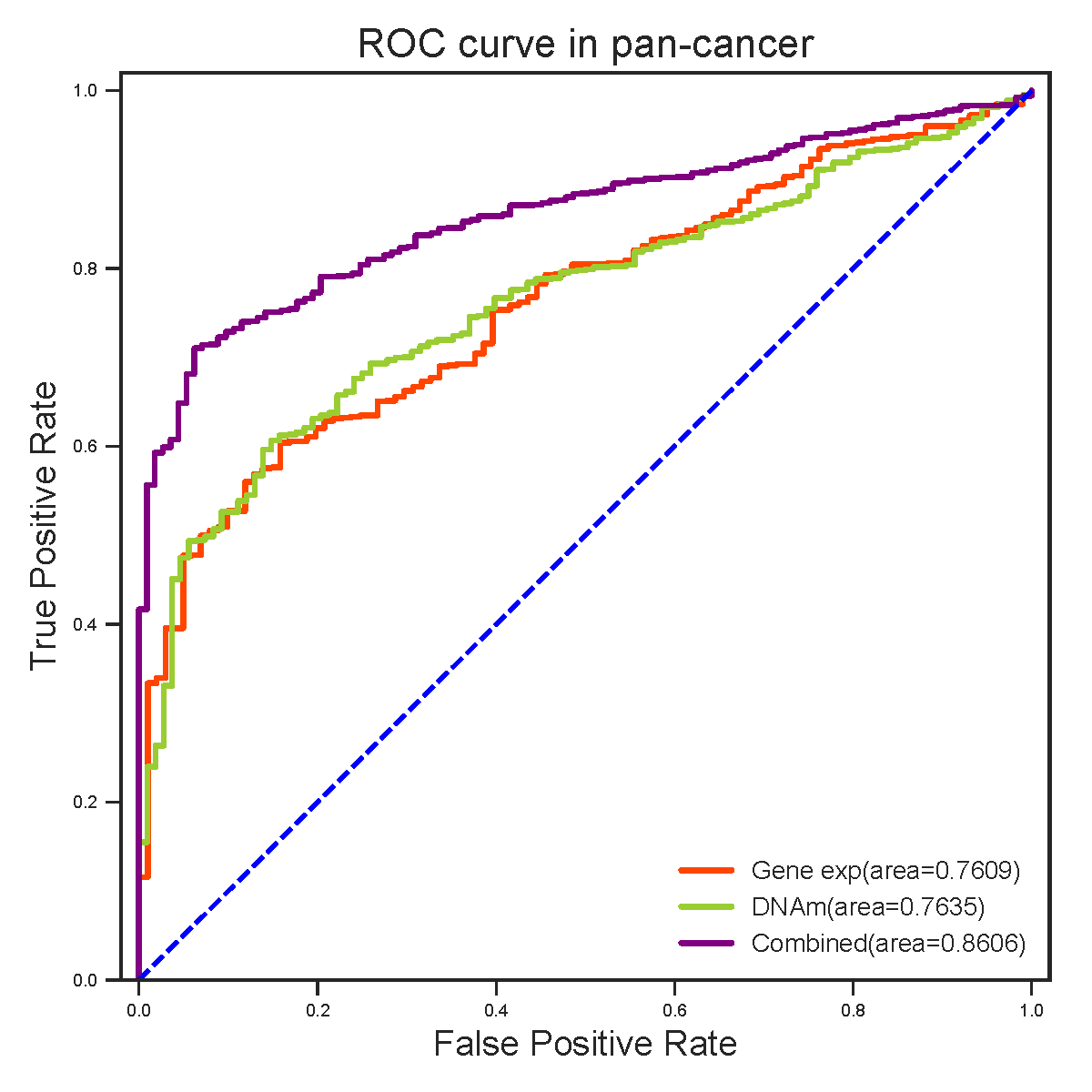


**Supplementary Figure 2.** Six Genes in zinc fingers gene family contribute to cancer diagnosis. ROC curve of diagnosis module constructed from gene expression profile, DNA methylation profile or combined gene expression and DNA methylation level of the 6 genes. Logistic regression module was implemented in this diagnosis model.

**
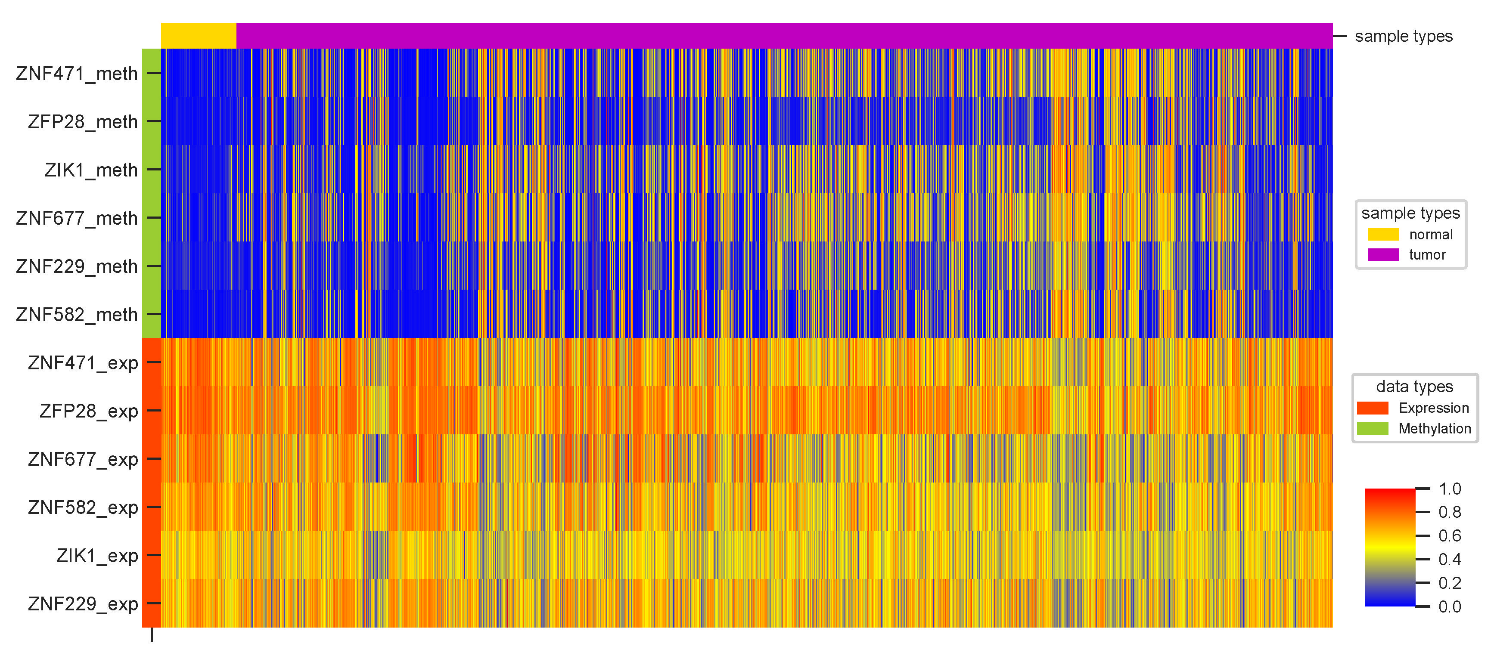
**

**Supplementary Figure 3.** Clustermap of expression and DNA methylation profile of six genes in zinc fingers gene family in 14 cancer types. Row colors indicate gene expression and DNA methylation data type, and col colors represent tumor or normal samples from 14 cancers (5519 samples in total). DNA methylation and gene expression values were standard scaled to 0-1 by the row.

**
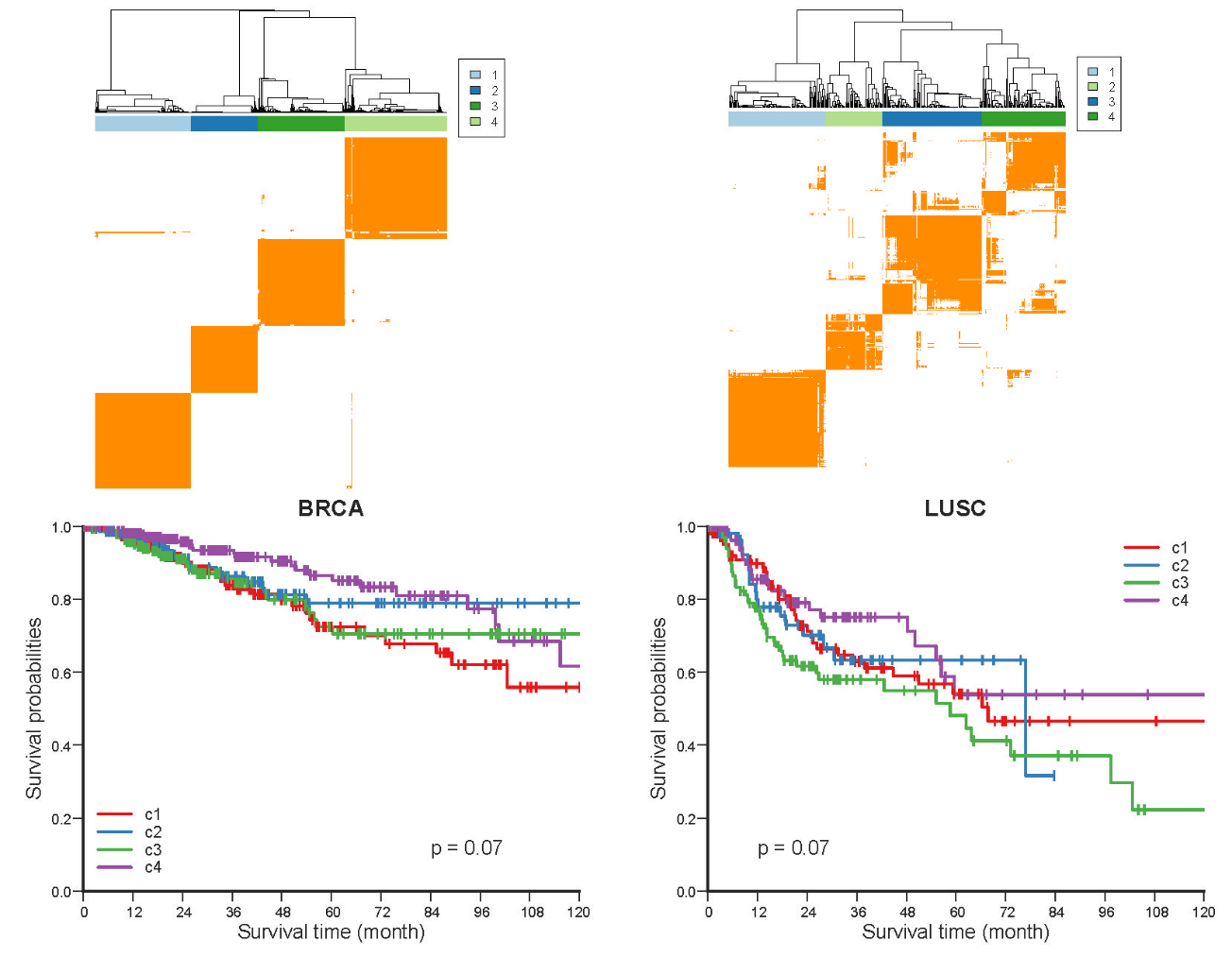
**

**Supplementary Figure 4.** Kaplan-Meier plot of PFI for distinct subtypes of BRCA and LUSC. Consensus cluster plot (top) and K-M survival plots (bottom) were separately shown for 8 disparate cancers.

## Supplementary Tables

Supplementary table 1 – Gene functional enrichment analysis result for COME events.

Supplementary table 2 – Enrichment analysis for the gens involved in functional epigenetic modules of the zinc finger gene family.

Supplementary table 3 - Frequently occurring COME events.

Supplementary table 4 – Statistics for the number of overlapping samples between COME subtypes and the origin or tumor tissues.

Supplementary table 5 - Number of samples overlapping between COME subtypes and cancer tissue of origin.
